# A new digital evaluation protocol applied in a retrospective analysis of periodontal plastic surgery of gingival recessions

**DOI:** 10.1038/s41598-021-99573-6

**Published:** 2021-10-14

**Authors:** Tiago Marques, N. M. Santos, Joana Fialho, J. Montero, A. Correia

**Affiliations:** 1 Faculty of Dental Medicine, Universidade Católica Portuguesa, Viseu, Portugal; 2 Centro de Estudos em Educação e Inovação (CI&DEI), Instituto Politécnico de Viseu, Viseu, Portugal; 3 Department of Surgery, Faculty of Medicine, University of Salamanca, Salamanca, Spain; 4 Faculty of Medicine, University of Salamanca, Salamanca, Spain; 5 Center for Interdisciplinary Research in Health, Universidade Católica Portuguesa, Viseu, Portugal; 6 Faculty of Dental Medicine, Universidade Católica Portuguesa, Estrada da Circunvalação, 3504-505 Viseu, Portugal

**Keywords:** Dentistry, Dental conditions, Periodontics

## Abstract

This research aimed to develop a new digital evaluation protocol to objectively quantify the volumetric changes of root coverage periodontal plastic surgery when combined with connective tissue graft. Consecutive patients with Cairo recession type 1 (RT1) or Cairo recession type 2 (RT2) were treated. Accurate study models obtained at baseline and follow-ups were optically scanned. Healing dynamics were measured by calculating volume differences between time points. Nineteen patients were treated between December 2014 and January 2019. At 3-month follow-up, root coverage was 95.6% (± 14.5%) with tunnel and connective tissue graft (TUN + CTG) technique, and 88.9% (± 20.5%) with the vestibular incision subperiosteal tunnel access and connective tissue graft (VISTA + CTG) technique. Recession decreased 1.33 (± 0.86) mm and 1.42 (± 0.92) mm, respectively (p = 0.337). At 6-month follow-up, root coverage was 96.5% (± 10.4%) with the TUN + CTG and 93.9% (± 10.3%) with the VISTA + CTG. Recession decreased 1.35 (± 0.85) mm and 1.45 (± 0.82) mm, respectively (p = 0.455). Complete root coverage was achieved in 86.7% (± 0.4%) with TUN + CTG and 70.6% (± 0.5%) with VISTA + CTG. No statistically significant differences were found between techniques. The digital protocol presented proved to be a non-invasive technique for accurate measurements of clinical outcomes. Both techniques reduce gingival recessions, with no statistically significant differences.

## Introduction

The development of intra-oral and laboratory scanners associated with 3D analysis software make it possible to evaluate volumetric changes in the hard and soft tissues of the oral cavity^1^. This technology was first described to measure in vitro alveolar ridge defects. It has been adopted in some experimental clinical studies to measure soft tissue and volumetric changes allowing the assessment of outcome interventions. Nonetheless no guidelines are implemented to standardize the assessment methods^1^. Concerning periodontology, there are publications in the dental literature that use optical scanning-based digital technologies, for evaluating volumetric changes following implant placement, soft tissue augmentation at implant sites, ridge augmentation, ridge preservation and root coverage procedures^2–6^.

Gingival recession is a condition that affects mostly adults when the root surfaces of one or more teeth are exposed due to an apical displacement of gingival tissues^7–10^. It can have several etiologies, which may be grouped into anatomical factors (e.g., lack of attached gingiva, muscular insertions near gingival margins, tooth misalignment, inadequate thickness of the alveolar bone plate, and root prominences), pathological conditions (e.g., periodontitis or viral infections), and iatrogenic factors (e.g., improper restorations within the biological space and mechanical trauma, including trauma associated with tooth brushing or lip piercing)^11^.

Multiple studies have documented simultaneous treatment of contiguous recession defects using large, partial-thickness, coronally advanced envelop flaps, often including connective tissue grafts^12,13^. The tunnel approach with connective tissue grafts keeps papillary integrity and avoids vertical releasing incisions, allowing the treatment of multiple contiguous recession defects^14^. Similarly, buccal recession coverage with free autogenous soft-tissue grafts of epithelium and connective tissue has also provided consistent clinical results^14–16^. These grafts success has been attributed to the double-blood supply at the recipient site from the underlying connective tissue base and the overlying recipient flap^17,18^.

However, the use of the intrasulcular approach to creating either a sub- or supraperiosteal space to extend beyond the mucogingival junction followed by the placement of a connective tissue graft is technically demanding^12^ and has several disadvantages, such as the risk of perforation or trauma of the sulcular tissues^12^, accidental papilla laceration, reduced coronal mobilization of the flap, reduced access for graft placement, and reduced papilla mobilization. The current techniques limitations also include scar formation at the recipient site resulting from surface incisions, yielding possible unfavorable healing outcomes^12,19^. On the other hand, it is assumed that avoiding visible incisions on the tissue surface allows for improved esthetics due to minimal soft-tissue trauma and post-operative scar tissue formation without complications during the healing phase^20,21^. Therefore, the vestibular incision subperiosteal tunnel access (VISTA) technique was developed to overcome some of the main drawbacks of other tunnel techniques reported in the literature^12^.

There is still some lack of knowledge about the tissue thickness over the denuded root. Very few studies^22^ have attempted to quantify soft-tissue thickness over the exposed root. Specifically, flap thickness has been shown to predict root coverage in mucogingival surgery^23,24^, with 1.1 mm being the minimum thickness to achieve complete root coverage. However, there is still a lack of information about the assessment of periodontal biotype changes and volume gain after a connective tissue graft.

Therefore, this research aimed to develop and present a new digital evaluation protocol to objectively quantify the volumetric changes of root coverage periodontal plastic surgery, particularly the tunnel and VISTA techniques combined with connective tissue graft, with a 3- and 6-month follow-up. The null hypothesis established was: there is no difference on root coverage outcomes when applying two different periodontal plastic surgical techniques.

## Results

Nineteen patients (4 males and 15 females) treated between December 2014 and January 2019 were enrolled in this study. Their mean age was 26.4 (± 7.9) years (age range, 17–48 years). One patient had recessions on anatomically separate locations of the maxilla, which derived in two independent surgical sites. A total of 38 recessions were treated for scientific evaluation. However, three patients from the Tunnel group did not attend the 6-month follow-up, and thus, only 32 defects were evaluated at 6 months, 15 tunnel and 17 VISTA. The TUN + CTG group contributed with 21 defects, and the VISTA + CTG group with 17 defects. Details regarding patients characteristics at baseline are shown in Tables 1 and 2. No complications occurred in any included patients during the follow-up period.

**Table 1:** Patients’ and site-specific characteristics for the tunneling technique.

Patient ID	Age (years)	Gender	Tooth	Recession	CEJ	Step(+/−)	Surgical Approach
01	38	Male	16	RT1	Undetectable	+	Tunnel
			15	RT1	Detectable	+	
			14	RT1	Undetectable	+	
02	26	Female	23	RT1	Detectable	+	Tunnel
03	24	Female	13	RT1	Detectable	−	Tunnel
			14	RT1	Detectable	−	
			16	RT1	Detectable	−	
04	24	Male	34	RT1	Detectable	−	Tunnel
			35	RT1	Detectable	−	
05	23	Female	35	RT1	Undetectable	+	Tunnel
06	21	Female	31	RT1	Detectable	−	Tunnel
07	26	Female	14	RT1	Detectable	−	Tunnel
			24	RT1	Detectable	−	
08	29	Female	34	RT1	Undetectable	+	Tunnel
			35	RT1	Detectable	−	
			36	RT1	Undetectable	+	
09	20	Female	16	RT1	Detectable	−	Tunnel
			15	RT1	Detectable	−	
			14	RT1	Detectable	−	
10	24	Female	24	RT1	Detectable	−	Tunnel
			25	RT1	Detectable	−	

*Recession* Cairo’s recession classification, *CEJ* cementoenamel junction, *Class A* detectable CEJ, *Class B* undetectable CEJ, *Step* root surface concavity, + presence of a cervical step, − absence of a cervical step.

**Table 2:** Patients’ and site-specific characteristics for the VISTA technique.

Patient ID	Age (years)	Gender	Tooth	Recession	CEJ	Step (+/−)	Surgical Approach
11	23	Male	21	RT2	Detectable	−	VISTA
12	21	Male	41	RT2	Detectable	−	VISTA
13	17	Female	41	RT1	Detectable	−	VISTA
14	28	Female	43	RT1	Detectable	−	VISTA
15	42	Female	16	RT1	Undetectable	+	VISTA
			15	RT1	Undetectable	+	
			14	RT1	Detectable	−	
16	22	Female	31	RT2	Detectable	−	VISTA
			32	RT2	Detectable	−	
			41	RT2	Detectable	−	
			42	RT2	Detectable	−	
17	22	Female	31	RT1	Detectable	−	VISTA
18	48	Female	24	RT2	Undetectable	+	VISTA
			25	RT1	Detectable	−	
19	23	Female	43	RT1	Detectable	−	VISTA
			44	RT1	Detectable	−	
			45	RT1	Detectable	−	

*Recession* Cairo’s recession classification, *CEJ* cementoenamel junction, *Class A* detectable CEJ, *Class B* undetectable CEJ, *Step* root surface concavity, + presence of a cervical step, − absence of a cervical step.

The superimposition was performed, and the results obtained showed mostly green surface which indicates both the study model and reference model corresponded to one another. Using the best fit algorithm, we obtained a more precise alignment minimizing the differences between the surfaces, this precision is translated by the Root mean square value (RMS) t shows how accurate the process is. A higher calculated RMS value indicated a large error, i.e., the difference in the attributes between reference and measurement data. RMS mean value for the 0–3 months scans was 0.14 (± 0.05) mm and 3 to 6 months was 0.12 (± 0.03) mm as shown on Supplementary Table 2.

### Gingival recession characteristics

Recession depth, recession area, and gingival margin thickness are described in Table 3. In the TUN + CTG group, ten experimental sites were RT1: a total of 21 recessions, including three cases of single-defect recessions and seven multiple-defect recessions. Mean baseline recession depth was 1.38 (± 0.29) mm. The VISTA + CTG group included RT1 and RT2 recession types: a total of 17 recessions, including five cases of single-defect recessions and four multiple-defect recessions. Mean baseline recession depth was 1.60 (± 1.02) mm. A statistically significant reduction of the recession area was observed at both 3 months and 6 months (p < 0.001 in both), independently of the technique used. No statistically significant changes were observed in the recession area between the 3- and 6-month follow-ups (p = 0.613) on tunnel (p-value = 0.317) and VISTA (p-value = 0.893) respectively. No statistically significant changes were observed in the mean gingival thickness and maximum gingival thickness in 2D or 3D (2DTHK, 2DTHKMax, 3DTHK, 3DTHKMax).

**Table 3:** Recession depth and marginal soft-tissue thickness (2DTHK, 2DTHK Max, 3DTHK, 3DTHK Max mm) at baseline, 3 months, and 6 months.

	TUNNEL + CTG		VISTA + CTG		*p*-value
	Recession depth				
	n	Mean ± SD (mm)	n	Mean ± SD (mm)	
Baseline	21	1.38 ± 0.29	17	1.60 ± 1.02	0.601
0–3 months	21	0.05 ± 0.16	17	0.17 ± 0.29	0.322
3–6 months	15	0.04 ± 0.11	17	0.14 ± 0.30	0.433

	Recession area				*p*-value
	n	Mean ± SD (mm^2^)	n	Mean ± SD (mm^2^)	
Baseline	21	4.23 ± 0.83	17	5.12 ± 6.07	0.561
0–3 months	21	0.25 ± 0.81	17	0.86 ± 2.11	0.352
3–6 months	15	0.25 ± 0.75	17	1.10 ± 3.15	0.433

	Gingival margin thickness				*p*-value
	n	Mean ± SD (mm)	n	Mean ± SD (mm)	
Baseline	21	0.78 ± 0.34	17	0.50 ± 0.24	**0.000**

	2D mean gingival thickness (2DTHK)				*p*-value
0–3 months	21	0.53 ± 0.28	17	0.54 ± 0.32	0.916
3–6 months	15	0.00 ± 0.18	17	0.01 ± 0.13	0.870

	2D maximum gingival thickness (2DTHK Max)				*p*-value
	n	Mean ± SD (mm)	n	Mean ± SD (mm)	
0–3 months	21	1.20 ± 0.39	17	1.18 ± 0.48	0.902
3–6 months	15	0.43 ± 0.43	17	0.43 ± 0.26	0.982

	3D mean thickness of tissue over denuded root (3DTHK)				*p*-value
	n	Mean ± SD (mm)	n	Mean ± SD (mm)	
3 months	21	0.34 ± 0.13	17	0.32 ± 0.16	0.693
6 months	15	0.34 ± 0.14	17	0.33 ± 0.16	0. 813

	3D maximum thickness of tissue over denuded root (3DTHK Max)				*p*-value
	n	Mean ± SD (mm)	n	Mean ± SD (mm)	
3 months	21	1.19 ± 0.36	17	1.20 ± 0.46	0.968
6 months	15	1.10 ± 0.34	17	1.29 ± 0.47	0. 195

At the 3-month follow-up, root coverage was 95.6% (± 14.5%) for the TUN + CTG Group and 88.9% (± 20.5%) for the VISTA + CTG Group, with a recession reduction of 1.33 (± 0.86) mm and 1.42 (± 0.92) mm, respectively (p = 0.337). At the 6-month follow-up, root coverage was 96.5% (± 10.4%) for the TUN + CTG Group and 93.9% (± 10.3%) for the VISTA + CTG Group, with a mean recession reduction of 1.35 (± 0.85) mm and 1.45 (± 0.82) mm, respectively (p = 0.455). Complete root coverage was detected at 6 months in 86.7% (± 0.4%) of the TUN + CTG Group and 70.6% (± 0.5%) of the VISTA + CTG Group. No statistically significant differences were found between techniques (Table 4).

**Table 4:** Comparison of the Tunnel and VISTA Groups regarding recession depth reduction, percentage of root coverage, and percentage of defects with complete root coverage, at 3 and 6 months after surgery.

	Baseline: 3 months			Baseline: 6 months		
	Tunnel	VISTA	*p*-value	Tunnel	VISTA	*p*-value
Recession depth Reduction (mm)	1.33 ± 0.86	1.42 ± 0.92	0.931	1.35 ± 0.85	1.45 ± 0.82	0.706
% Root coverage	95.6 ± 14.5	88.9 ± 20.5	0.337	96.5 ± 10.4	93.9 ± 10.3	0.455
Area reduction (mm^2^)	3.98 ± 2.49	4.26 ± 4.47	0.576	3.88 ± 2.29	4.02 ± 3.73	0.467
% Defects with complete root coverage	90.5 ± 0.3	70.5 ± 0.5	0.308	86.7 ± 0.4	70.6 ± 0.5	0.455

**Figure 1:**
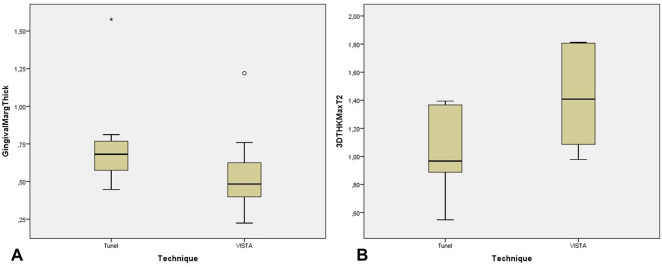
(**A**) Box-plot showing the gingival margin thickness (mean and interquartile range) of patients treated with the tunnel vs. VISTA techniques. Symbols (star and circle) indicate outliers of the study sample. (**B**) Box-plot showing 3DTHKMax T2 (mean and interquartile range) for patients treated with the tunnel vs. VISTA techniques.

There was no significant thickness gain (2DTHK) between the 3-month and 6-month follow-ups (p = 0.778). Using the Mann–Whitney test to verify if the technique influences any variables, we found a statistically significant difference in the gingival margin thickness between the techniques (p < 0.001), confirming the surgeon’s technique choice according to the gingival biotype, where the tunnel technique was chosen for thicker biotypes (Fig. 1A). Even though VISTA technique shows a higher maximum thickness gained at 6 months there are no statistically significant differences (3DTHK Max) (p = 0.056) (Fig. 1B).

The measured data indicate a certain soft-tissue thickness above which no further benefit was obtained in terms of surgical outcome. A logistic model was calculated with the complete root coverage as the target variable and the mean marginal soft-tissue thickness at 6 months as the covariable (3DTHK). This analysis indicated that a mean 3DTHK of 0.35 mm was necessary to predict a complete root coverage with 95% confidence.

### Correlation between gingival margin thickness, papilla height, recession reduction area, and root coverage

A positive correlation was found between the gingival margin thickness and the recession reduction area at the 6-month follow-up (r = − 0.407; p = 0.025). On the other hand, the gingival margin thickness was not correlated with Cairo’s recession classification at 6 months (p = 0.77) or root coverage at both 3 months (p = 0.560) and 6 months (p = 0.531).

The papilla height was correlated with the recession reduction area at 6 months (r = − 0.636; p < 0.001) and 3 months (r = − 0.626; p < 0.001). However, the papilla height was not correlated with Cairo’s recession classification (p = 0.531) or the technique (p = 0.549). Also, no correlation was found between the papilla height and root coverage at both 3 months (p = 0.698) and 6 months (p = 0.315).

### Healing dynamics of treated sites

The results of the volumetric assessments performed are shown in Supplementary Table 3. The soft tissue gained at 3 months was regarded as the standard value of soft-tissue augmentation, the grafted sites showed a mean gain at 3 months of 4.45 (± 3.42) mm^3^ in the TUN + CTG Group and 5.27 (± 5.79) mm^3^ in the VISTA + CTG Group (p = 0.772). At 6 months, the treated sites showed a mean gain of 4.72 (± 3.88) mm^3^ in the TUN + CTG Group and 5.43 (± 5.21) mm^3^ in the VISTA + CTG Group (p = 1.000). From T1 to T2 (6 months), there was a mean volume increase of 13.2% (± 5%), where the TUN + CTG Group showed a higher mean volume increase of 18.9% ± 58.4%) and the VISTA + CTG Group of 8.1% (± 53.1%) (p = 0.710).

**Figure 2:**
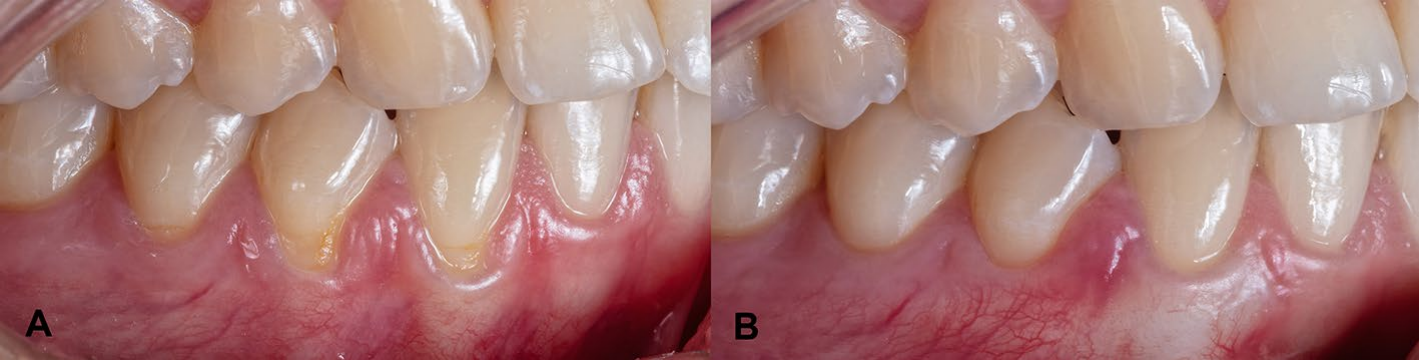
(**A**) Baseline, (**B**) 6 months results.

The clinical outcomes can be observed in Fig. 2A and B.

## Discussion

The digital evaluation protocol presented in this research is innovative as it adapts commonly measurements tools of a computer-aided-design software available mainly for engineering issues for a periodontology research in a way that was not found in the published literature, particularly in the analysis of variables like gingival margin thickness. In this research, a highly precise measurement method was used to evaluate the clinical outcomes of two different periodontal plastic surgical techniques in the treatment of gingival recessions. Within the limitations of this research, particularly related to the reduced sample size, the null hypothesis was not rejected, since no statistically significant differences were found in the clinical outcomes.

This research was done following the normal clinical environment of the University Dental Clinic. There was a full control of techniques and materials available for the surgical procedure to achieve the expected treatment success. The surgeon (T.M) had no knowledge that the presented research was being developed. Then, all the impressions were done by the author (N.S.) in a conventional way with alginate and cast models were obtained, as it were the commonly available dental materials. Although alginate has the lowest accuracy of all conventional impression materials, the use of regular casts that could include artifacts as those occurring in everyday practice offers a true representation of current clinical conditions^25^. Naturally, it could also account for part of the differences detected by different techniques, but it would do so in all cases, since the same methodology was used in every single case. It would be preferable to obtain the data directly with an IOS, to avoid a possible bias of conventional impression materials, but the availability of the IOS in the university dental clinic was not possible in all the moments, reason why the methodology described before was adopted.

The use of intraoral scanners goes back to 1987 known as the Chairside Economical Restoration of Esthetic Ceramics^26^. They were firstly used to measure alveolar ridge defects^6^. Since then, it has been successfully adopted to mucogingival surgery^22,27–29^. The intra-oral scanner used to do the 3D reconstruction of the cast models in the University Dental Clinic is from the year 2015. Although there are currently new IOS with better characteristics, this scanner allow to capture a precise image in small dimension areas as those that we have done^30, 31^. It is not a IOS to perform a full-arch digitalization^32^.

Considering the surgery techniques in evaluation, both showed recession reduction and complete root coverage. TUN + CTG and VISTA + CTG had similar root coverage results VISTA + CTG (95.6% (± 14.5%) and 88.9% (± 20.5%), respectively), with no statistically significant differences. Our study showed a mean root coverage superior to the latest systematic review and it can be explained by the microsurgical approach and the split-thickness used in the TUN + CTG^33^. Complete root coverage at 6 months was also highest in the TUN + CTG Group with 86.7% (± 0.4%) than the VISTA + CTG Group, with 70.6% (± 0.5%). However, both these differences might be due to all Cairo RT2 recessions being in the VISTA + CTG group, reducing the percentage of root coverage in this group. On the other hand, the higher recession reduction in the VISTA + CTG group of 1.42 (± 0.92) mm vs. 1.33 (± 0.86) mm in the TUN + CTG group is probably explained by the bigger recessions in the VISTA + CTG group^14^.

The flap design is a key factor in the outcomes following root coverage and is considered a prognostic factor in the treatment of gingival recession defects^23,34,35^. Several studies have correlated greater flap thickness to better clinical outcomes after root coverage^36^. Also, a preoperative gingival marginal thickness greater than 1 mm correlates with a higher percentage of root coverage^28^. In our study, we described a unique non-invasive methodology to determine the pre-surgical gingival margin thickness. To the best of our knowledge a similar metrology technique was not found in previous published literature. The precise results obtained found a strong correlation with recession area reduction, which is in line with the literature. Gingival margin thickness can be used as a noninvasive surrogate for flap thickness measurement^37^.

The correlation between marginal soft-tissue thickness and root coverage stability is still debated because not many researchers have actually aimed to assess the soft-tissue thickness over the exposed root surface^22^. It is believed that any increase in thickness will show long-term stability^22,38^. However, by comparing baseline and following soft-tissue profiles and volume differences, we can determine the thickness of the marginal soft tissues that have been obtained over the denuded root. Evidence has shown that combining the procedure with an autologous CTG is the most effective and predictable surgical approach for covering gingival recession defects^38–40^.

The use of a de-epithelialized graft allows integration in the graft the connective tissue adjacent to the epithelium, which is denser, firmer, more stable, and supposedly more suitable for root coverage^41,42^. These types of CTGs demonstrate a greater increase of gingival thickness at the buccal aspect than subepithelial CTGs that lose a significant part of their thickness during the healing phase^41^. In our study, the amount of thickness gain needed for complete root coverage diverged considerably from the results by Zuhr et al.^27^ because of the type of connective tissue harvested. We used a de-epithelialized graft, which tends to be more stable in terms of contraction than subepithelial grafts. The different measuring techniques could also explain such a difference.

In our study, the creeping attachment phenomenon happened due to increased volumetric alterations after 6 months, which disagrees with others papers published^22,27^. These dissimilarities are probably explained by the different types of connective tissue graft used in this study. Both cited papers^22,27^ indicate a contraction of the graft, around two-thirds of the augmented volume was maintained after 12 months and soft-tissue healing was accomplished at 6 months. In our research, using a de-epithelized graft, we saw a mean volume increase after 6 months 13.2% (± 55%).

Our study’s measured data indicated a certain soft-tissue thickness above which no further benefit was seen regarding the surgical outcome. Therefore, a logistic model was calculated with the complete root coverage as the target variable and the mean marginal soft-tissue thickness at 6 months as the covariable (3DTHK). This analysis indicated that a mean 3DTHK of 0.35 mm had to be maintained for predicting a complete root coverage with a confidence of 95%. This finding is confirmed by some recommendations on clinical decision making that incentivize the usage of thin CTGs for root coverage^38,43^.

Volume changes and the healing process seems to be accomplished after 6 months^22^. We present new data indicating that grafted sites with de-epithelized grafts will experience a volume increase until the 6 months. However, due to this study’s limitations, a higher patient number would be necessary to draw any valid conclusions. We now intend to conduct a 2-year follow-up to evaluate the stability of these types of grafts. We recommend small-sized and thin grafts because these could enhance the nutritional exchange between the recipient site, graft, and covering flap.

The present retrospective study has some limitations, including being retrospective, a small sample size, and having no randomization of treatments, direct digital impressions couldn’t have been done in our study due to the availability of intra oral scans at the time the first patients were treated. Also, an esthetical evaluation could not be performed because of the retrospective characteristics of the analysis. This study is also limited in comparing the two techniques because thin biotypes and RT2 were treated with VISTA technique being the most suitable technique to avoid the risk of perforation or trauma of the sulcular tissues, papilla laceration, reduced coronal mobilization of the flap, and reduced papilla mobilization. The number of RT2 was higher in the VISTA group and could therefore influence the results. This study reports the 6-month outcomes of two techniques. The long-term stability regarding the increased gingival thickness is hypothetical.

Nonetheless, the used measuring method gives new insights in high-precision outcome evaluation after surgical recession treatment, ensuring the same region of interest is analyzed on all post-surgical time points, with an insignificant level of error and high reliability^5^. A new non-invasive method to measure the gingival marginal thickness was developed, and new outcome parameters that are not easily measured in the clinical setting were examined in this study. In future studies, a randomized control clinical trial should be done to compare both techniques digitally by analyzing the outcomes, using recently developed intra oral scanners, directly without conventional impressions. The technique currently taken as the gold standard is clearly invasive, too dependent on the individual operator, and calibrated in millimeters, unable to measure microns and gingival volume increase^5^.

We characterized the healing dynamics of the grafted sites and assess the influence of the thickness in the newly created soft tissues on the outcome of the surgical techniques. This metrology technology allows for an unheard precision in linear measurements as in volumetric measurements and influences the study’s outcomes. It cannot be compared with studies where the measurements are done using a periodontal probe, where a rounding error must be accepted.

The digital protocol here described can be applied in future research since it has several advantages: non-invasive, easiness, precise. One property of a new method of gingival volume assessment should be the discriminative capacity of distinct situation not only in pre-post assessment but also between comparable patients that were treated with distinct techniques. This non-invasive method has demonstrated to be able to capture significant pre-post difference and clinical relevant trends between both surgical techniques. Furthermore, it follows the evolution of digital technology in Dentistry, and results can be even more precise in a near future due to new IOS technologies being introduced by dental manufacturers. In summary, the applied metrology technology offers new insights in evaluating the outcomes after surgical recession treatment with the highest precision and accuracy with both techniques providing a reduction in gingival recessions and an increase in gingival thickness with no statistically significant differences. This promising method could be easily popularized mainly because there is no gold standard for non-invasive evaluations currently available.

## Methods

All the surgeries were performed by the same surgeon (T.M.), without knowing that this research was being developed with the focus of achieving the best patient clinical outcomes. The clinical criteria of the surgeon to choose one technique over the other was related to the gingiva biotype and the root coverage procedure to be performed, according to the literature on this issue: thin biotypes were allocated to the VISTA technique and thick biotypes to the TUN technique^12^. Only in 2020, the authors decided to do this research, as a retrospective study, so there has been no bias of the surgeon clinical performance concerned to this research.

The study protocol was approved by the Ethics Commission for Health of the University (Comissão de Ética para Saúde da UCP, Report number 25, 4^th^ of June 2020). Informed consent was obtained from all participants and all methods were performed in accordance with the Declaration of Helsinki principles for medical research involving human subjects and following the requirements established by Portuguese Law n.º 21/2014 for clinical research.

### Participants

All participants were selected among the patients that visited the Periodontology Area of the University’s Dental Clinic. Consecutive patients with no loss of interproximal attachment (Cairo recession type 1 (RT1)) or loss of interproximal attachment equal or not greater than the buccal attachment (Cairo RT2) treated in the University’s Clinic were enrolled in the study. Patients exhibiting single or multiple adjacent gingival recessions were treated, and all defects were included in the data collection. No single case was missed, overseen, or excluded. Interestingly, all Cairo RT2 recessions were included in the VISTA + CTG group.

Patients with one or more gingival recession defects who satisfied the following inclusion criteria were included:

- Periodontally and systemically healthy patients (for example, patients with ASA classifications I and II);
- Minimum of one Cairo RT1 or RT2 buccal or lingual gingival recession defect;
- Full-mouth plaque and bleeding scores ≤ 20%, no pocket depths > 3 mm, no active periodontal disease;
- Clinical indication and/or patient request for recession coverage;
- Radiographic evidence of sufficient interdental bone.

Teeth presenting root steps at the CEJ level and/or presence of root/crown abrasion and teeth presenting any sort of malpositioning were not excluded from the study. Exclusion criteria were the following:

- Cairo RT3 recession defects;
- Smokers;
- Teeth with cervical restorations
- Patients unable to undergo minor oral surgical procedures;
- Patients with a history of drug or alcohol abuse or psychiatric disorder;
- Pregnant patients;
- Uncontrolled periodontal disease or patient’s unwillingness to undergo needed periodontal therapy around remaining teeth;
- Patients who had any systemic condition that could contraindicate any other surgical procedures.

#### Study

This research was designed as a retrospective cohort study. A post-hoc statistical analysis indicates that this study would require a sample size of 102 for each group (i.e. a total sample size of 204, assuming equal group sizes), to achieve a power of 80% and a level of significance of 5% (two sided). However, the periodontal plastic surgeries performed in the University Dental Clinic in the time-frame of this research were fewer, allowing to record data from 19 patients and 38 recessions (31 RT1 and seven RT2). With this reduced sample size, the post-hoc power was only 8%. Hence, this was considered a pilot research where a new digital evaluation protocol was used to objectively quantify the volumetric changes of root coverage periodontal plastic surgery techniques.

The clinical outcomes were digitally evaluated by a different researcher (N.M.S.), blinded to the treatment technique. This researcher did not know the case and the technique used. A number was assigned to each case, not having any kind of identification.

Using an intraoral scanner (Intraoral Scanner DentalWings; Straumann, Basel, Switzerland), at baseline, 3 months, and 6 months, the scans were performed by the same operator (N.M.S.), following the manufacturers scanning protocol limited to the quadrant or sextant affected by the gingival recessions.

### Pre-surgical preparations

Periodontal basic therapy was performed in all participants before surgery, included oral hygiene instructions and motivation, dental prophylaxis, and low-abrasive air polishing (Perio-Mate; NSK, Eschborn, Germany) as a mean of plaque removal^44^. Patients were required to use an atraumatic brushing technique with a soft toothbrush (Elgydium Clinic 7/100; Pierre Fabre Oral Care, Paris, France) or electrical toothbrush (OralB Smart 1500 Electric Rechargeable Toothbrush; Procter & Gamble, Cincinnati, Ohio, EUA) to eradicate harmful habits associated with the gingival recessions.

### Surgical protocol

The tunnel approach was performed basically according to Zuhr et al.’s (2007) descriptions of a modified microsurgical tunnel technique^22,45,46^.

Following an initial sulcular incision with a microsurgical blade (SB004/BW064; MJK, Marseille, France), tunneling knives and blades were used to create a split-thickness flap, to develop a continuous tunnel in the buccal or lingual soft tissues. The supra-periosteal dissection was extended well into the mucosal tissues for sufficient flap mobility. Papillae were carefully detached by a full-thickness preparation in their buccal aspect, thus allowing for a coronal displacement of the mobilized buccal soft-tissue complex^22^.

The VISTA + CTG technique was performed according to Zadeh et al.’s descriptions of a modified microsurgical tunnel technique^12^.

Following local anesthesia, root planning of the exposed root surface was performed with Gracey curettes (LM; LM-Instruments Oy, Parainen, Finland). Then, the residues were removed by copious irrigation with a sterile saline solution. The VISTA approach was performed with a mucosal buccal access incision mesial to the recession defects being treated. Mucosal and intrasulcular incisions around the involved tooth were performed using microsurgical blades and specially designed tunneling knives (Tunneling Kit; Deppeler, Role, Switzerland), creating a mucoperiosteal tunnel exposing the facial osseous plate and root dehiscence. This tunnel was extended at least one or two teeth beyond the ones requiring root coverage to mobilize gingival margins and facilitate coronal repositioning, employing the same tunneling knives. Additionally, the subperiosteal tunnel was extended interproximally under each papilla as far as the embrasure space allowed, without making any surface incisions through the papilla. Finally, to achieve complete mobilization of the flap, the interdental papillae were gently undermined using a specially designed tunneling knife (Tunneling instrument TKP; Deppeler, Role, Switzerland)^18^.

The surgeon chose a de-epithelization approach of the posterior palate for CTG harvesting. The graft was thinned to a thickness of 1–1.5 mm and then slide into the previously created tunnel using a monofilament suture (Dafilon 6/0; BBraun, Melsungen, Germany).

### Post-surgical protocol

The post-surgical protocol was adapted from Zuhr et al.^45^. The patients received 600 mg of ibuprofen (Spidifen 600; Zambon, Bresso, Italy) after the surgical procedure to reduce swelling and were instructed to avoid any mechanical trauma in the surgical site for 2 weeks.

Patients were instructed to rinse with Chlorhexidine digluconate 0.12% (Eluperio; Pierre Fabre Oral Care, Paris, France), three times per day for 2 weeks.

Two weeks after surgery, patients started to clean the teeth in the post-surgical area with a soft brush or electrical toothbrush. Every patient was recalled 3 months and 6 months post-surgery when clinical data were recorded, and accurate casts were obtained.

### Digital measurements at baseline, 3 months, and 6 months

Accurate models were obtained at baseline and succeeding follow-up examinations, always under the same protocol: alginate impressions (Orthoprint; Zhermack, Badia Polesine, Italy) and gypsum study models (Vel-Mix white die stone; KERR, Bioggio, Switzerland), according to the manufacturer’s instructions. This methodology was based on the publications of Gil et al.^28,29^.

These models were optically scanned with (Intraoral Scanner DentalWings; Straumann, Basel, Switzerland), that has an accuracy of 20 μm (single unit), 50 μm (full arch) according to Dental Wings testing standard^47^.

A new methodology that uses computerized measurement tools and design software was applied with (Geomagic Control X; Geomagic, Morrisville, EUA) and (Magics 23 Materialise; Materialise, Leuven, Belgium). This software were used to virtually superimpose each clinical case’s pre-operatory 3D images subsequential follow-up scans and match them into one common coordinate system. All digital measurements were recorded to the nearest 0.01 mm as in Zuhr et al.^27^. The tooth surfaces were used as reference points for superimposing the different optically acquired files, allowing for accurate assessment of soft-tissue profile over the 6 months. A final alignment was done through the best-fit alignment algorithm^24^.

(A further description of the digital protocol has been included in Supplementary Table 1 to share with clinician and researchers the major steps of this digital assessment.)

The following variables were analyzed fully digitally with the before mentioned software:

- Gingival margin thickness (baseline);
- Recession depth measured from CEJ to the gingival margin at the central buccal site;
- Area of the gingival recession;
- Height of the gingival margin papillae;
- Recession reduction;
- Area reduction;
- Complete root coverage;
- 2D mean gingival thickness gain at 3 months and 6 months (2DTHK);
- 2D maximum gingival thickness gain at 3 months and 6 months (2DTHK Max);
- Volume over the denuded root at 3 months and 6 months;
- 3D mean thickness of tissue over the denuded root at 3 months and 6 months (3DTHK);
- 3D maximum thickness of tissue over the denuded root at 3 months and 6 months (3DTHK Max).

**Figure 3:**
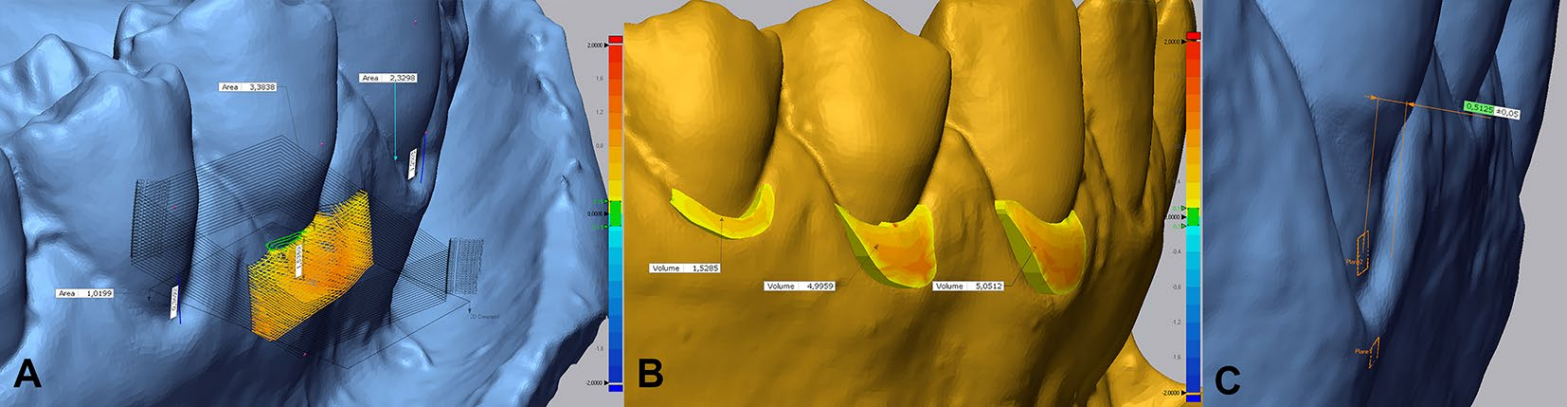
(**A**) Insertion of section plans perpendicular to ROI’s entire length. (**B**) The volume of tissue over the denuded root obtained after connective tissue graft between T0 (baseline) and T2 (6 months). (**C**) Two planes are defined, and the distance between them is automatically calculated.

Two different methods to measure the soft-tissue thickness were applied using the protocol presented in Supplementary Table 1. The first method calculated the mean gingival thickness (2DTHK) and maximum gingival thickness (2DTHK Max) for each case at every time point, as seen in Fig. 3A. The second method involved a 3D assessment by limiting the recession in T0, T1, and T2 at the same time using transparencies to obtain the new volume of tissue over the denuded root for precise quantification of the tissue, as seen in Fig. 3B. In this case, the region of interest comprised the entire area of grafted soft tissue on the previously exposed root surface. Thus, the mean thickness of the marginal soft tissue (3DTHK) on the formerly exposed root surface, the mean gingival thickness (3DTHK), and maximum gingival thickness (3DTHKMax) were assessed for each case and each time point. Gingival margin thickness was measured using a new method that allows quantifying the margin thickness in a non-invasive way, as shown in Fig. 3C: two planes are defined, and the software calculates the distance between those two planes.

### Statistical analysis

A dedicated computer software (Statistical Package for the Social Sciences, v24.0; IBM, Armonk, NY, USA) was utilized to carry out data analysis. Descriptive statistics were performed using mean values, standard deviations, frequencies, and percentages. Mean values were calculated from the parameters measured at the recession sites at different time points.

Differences were considered statistically significant at p < 0.05. Normal distributions were checked by Shaphiro-Wilk Test, however, given the small sample size of each group, the Mann–Whitney test (non-parametric) was used instead of the paired T tests. All hypothesis tests were conducted at the 5% level of significance.

## Supplementary Information

Supplementary Information 1.

Supplementary Information 2.

Supplementary Information 3.
