# Supplementary material for: A new digital evaluation protocol applied in a retrospective analysis of periodontal plastic surgery of gingival recessions
Source: Sci Rep. 2021 Oct 14;11:20399. doi: 10.1038/s41598-021-99573-6 (PMC8516885; doi:10.1038/s41598-021-99573-6)
Supplement: Supplementary file 1 — Supplementary Information 1. [file 41598_2021_99573_MOESM1_ESM.docx]

| **Protocol for the digital analysis’s 3D measurements** |
| --- |
| ***Geomagic® software*** |
| ***3D Compare function***: creates a map of colors (from +2 mm to -2 mm, with a tolerance of ± 0.2 mm) to evaluate the volumetric changes that occurred in the surgical area. |
| ***Spline function***: a coronal section was defined through the cementoenamel junction and mucogingival midline points, and several perpendicular planes with 0.1 mm between them were created. These perpendicular planes’ mesial-distal distance corresponded to the mesial-distal tooth width. In the intersection of these two planes, standardized points were created in each case to evaluate the tissue thickness covering the previously denuded root. |
| **This software allows saving the coordinates of the previously measured points so that the same measurements are applied in the following recalls in a precise and reproducible way.** |
| ***Multiple 2D Compare function***: creates a rectangular section standardized for each root coverage case, starting at the mucogingival line and ending at the cementoenamel junction. These limits were used to determine the region of interest for the measurements at the different time points, to perform an accurate and rigorous evaluation. |
| **Magics 23 Materialise® software** |
| STL files → mesh surface converted to a solid model → boolean operation T1 – T0 → solid model representing volume changes. |
| The recession was delineated at T0, T1, and T2 at the same time using transparencies, and the new volume of tissue over the denuded root was obtained, allowing precise quantification of the tissue. |

**Supplementary Table 1. Protocol for digital analysis.**
