# Supplementary material for: A new digital evaluation protocol applied in a retrospective analysis of periodontal plastic surgery of gingival recessions
Source: Sci Rep. 2021 Oct 14;11:20399. doi: 10.1038/s41598-021-99573-6 (PMC8516885; doi:10.1038/s41598-021-99573-6)
Supplement: Supplementary file 2 — Supplementary Information 2. [file 41598_2021_99573_MOESM2_ESM.docx]

| **PatientID** | | **RMS Best Fit alignment** | |
| --- | --- | --- | --- |
|  |  | 0-3 Months (mm) | 3-6 Months (mm) |
| **Tunel** | 1 | 0,17 | 0,1195 |
|  | 2 | 0,1244 | 0,2015 |
|  | 3 | 0,1179 | 0,113 |
|  | 4 | 0,138 | NC |
|  | 5 | 0,0935 | NC |
|  | 6 | 0,1187 | 0,0871 |
|  | 7 | 0,0804 | 0,0682 |
|  | 8 | 0,0778 | 0,1067 |
|  | 9 | 0,2601 | NC |
|  | 10 | 0,146 | 0,1489 |
| **VISTA** | 11 | 0,1825 | 0,1016 |
|  | 12 | 0,1283 | 0,0899 |
|  | 13 | 0,1755 | 0,0955 |
|  | 14 | 0,0875 | 0,0964 |
|  | 15 | 0,1755 | 0,1037 |
|  | 16 | 0,2484 | 0,1438 |
|  | 17 | 0,106 | 0,1034 |
|  | 18 | 0,1253 | 0,1417 |
|  | 19 | 0,1088 | 0,1184 |
|  | **Mean** | **0,140±0.051 mm** | **0,115±0.031 mm** |

**Supplementary Table 2. Alignment accuracy, root mean square value (RMS)**
