# Supplementary material for: A new digital evaluation protocol applied in a retrospective analysis of periodontal plastic surgery of gingival recessions
Source: Sci Rep. 2021 Oct 14;11:20399. doi: 10.1038/s41598-021-99573-6 (PMC8516885; doi:10.1038/s41598-021-99573-6)
Supplement: Supplementary file 3 — Supplementary Information 3. [file 41598_2021_99573_MOESM3_ESM.docx]

|  |  | **Volume Alterations at 3 months** | | **Volume Alterations at 6 months** | |
| --- | --- | --- | --- | --- | --- |
| **Patient ID** | **Tooth** | **Absolute** | **Relative** | **Absolute** | **Relative** |
| **01** | 16 | 2.78 | 100 | 2.79 | 0.41 |
|  | 15 | 2.51 | 100 | 2.95 | 17.66 |
|  | 14 | 4.43 | 100 | 4.86 | 9.67 |
| **02** | 23 | 9.56 | 100 | 11.30 | 18.17 |
| **03** | 13 | 6.92 | 100 | 7.86 | 13.69 |
|  | 14 | 2.92 | 100 | 4.73 | 62.23 |
|  | 16 | 1.45 | 100 | 2.01 | 38.49 |
| **04** | 34 | 4.57 | 100 |  |  |
|  | 35 | 2.75 | 100 |  |  |
| **05** | 35 | 5.50 | 100 |  |  |
| **06** | 31 | 0.28 | 100 | 0.76 | 175.97 |
| **07** | 14 | 2.67 | 100 | 2.92 | 9.09 |
|  | 24 | 0.80 | 100 | 1.51 | 90.03 |
| **08** | 34 | 7.49 | 100 | 6.93 | -7.46 |
|  | 35 | 6.45 | 100 | 5.22 | -19.09 |
|  | 36 | 15.40 | 100 | 14.04 | -8.81 |
| **09** | 16 | 3.11 | 100 |  |  |
|  | 15 | 1.94 | 100 |  |  |
|  | 14 | 5.41 | 100 |  |  |
| **10** | 24 | 4.16 | 100 | 2.21 | -46.75 |
|  | 25 | 2.40 | 100 | 0.72 | -70.05 |
| **11** | 21 | 22.41 | 100 | 18.02 | -19.59 |
| **12** | 41 | 8.80 | 100 | 9.18 | 4.38 |
| **13** | 41 | 6.03 | 100 | 5.71 | -5.26 |
| **14** | 43 | 4.44 | 100 | 6.09 | 37.13 |
| **15** | 16 | 11.18 | 100 | 13.78 | 23.23 |
|  | 15 | 5.92 | 100 | 6.15 | 3.80 |
|  | 14 | 1.70 | 100 | 1.93 | 13.37 |
| **16** | 31 | 2.22 | 100 | 2.59 | 16.48 |
|  | 32 | 0.74 | 100 | 1.88 | 153.82 |
|  | 41 | 0.66 | 100 | 0.20 | -69.11 |
|  | 42 | 0.02 | 100 | 0.00 | -97.01 |
| **17** | 31 | 0.74 | 100 | 1.23 | 66.18 |
| **18** | 24 | 12.30 | 100 | 12.32 | 0.21 |
|  | 25 | 1.02 | 100 | 1.20 | 17.29 |
| **19** | 43 | 5.05 | 100 | 5.85 | 15.74 |
|  | 44 | 4.86 | 100 | 5.00 | 2.84 |
|  | 45 | 1.53 | 100 | 1.15 | -25.08 |
| **Mean** |  | **4.82±4.58** | **100%±0** | **5.10±4.58** | **13.18%±54.97** |

**Supplementary Table 3. Descriptive statistics with absolute (mm3) and relative (%) values of volume alterations following CT grafting at both the VISTA and TUN groups.**
